# Anal Fistula That Developed After Bilateral Vasectomy Complicated by Scrotal and Perineal Infections

**DOI:** 10.7759/cureus.80876

**Published:** 2025-03-20

**Authors:** Adam Kemp, Kristina Guyton, Moshe Wald

**Affiliations:** 1 Department of Urology, University of Iowa Hospitals and Clinics, Iowa City, USA; 2 Department of Surgery, University of Iowa Hospitals and Clinics, Iowa City, USA

**Keywords:** anal fistula, complications, discharge, perineum, vasectomy

## Abstract

We present a case of an anal fistula that developed after bilateral vasectomy complicated by scrotal and perineal infections. The anatomical location of this post-vasectomy fistula was unique, as previously reported vasocutaneous fistulas after vasectomy typically occur in the scrotum. Another unusual aspect of this case was that it developed in an otherwise healthy individual, without any predisposing risk factors such as prior genitourinary surgery, radiation, or voiding dysfunction. A repeat pelvic MRI did not identify this fistula, which was ultimately diagnosed through examination under anesthesia in the operating room and successfully treated with anal fistulotomy.

## Introduction

Infection is one of the most common complications following vasectomy. The rate of infectious complications following vasectomy varies depending on the surgeon's experience, technique, and definition but is cited as 1-2% in the most recent American Urological Association (AUA) vasectomy guidelines [1]. The most common infections following vasectomy include wound, urinary, and epididymal infections [2]. Variations in surgical technique and hematoma formation have been shown to be risk factors for infection [2]. Although there are limited studies describing the rate of post-vasectomy infections for specific comorbidities, the AUA guidelines on vasectomy reference Campbell's, Walsh's, and Wein’s general patient-specific risk factors for infection, which include advanced age, poor nutrition, smoking, chronic steroid use, immunodeficiency, distant coexistent infection, and colonized exogenous/endogenous material [3]. The majority of these infections are self-limited and resolve with a course of oral antibiotics, although case reports have described more significant infections, including Fournier’s gangrene, syphilis chancre formation, and endocarditis requiring valve reconstruction following vasectomy [4-6].

Fistula formation following vasectomy is a rare late complication. A case series in 1992 described 16 cases of cutaneous fistulas to the vas deferens over the previous 70 years [7]. A more recent review of rare complications following vasectomy in 2023 described cases of vasocutaneous fistula that involved reflux of urine through the ejaculatory duct, seminal vesicles, and the vas deferens, draining urine through the scrotal skin [8]. These fistulas occurred in patients who had recently undergone transurethral resection of the prostate (TURP) or had underlying voiding dysfunction secondary to neurogenic bladder or bladder outlet obstruction. In a case described in 2009, semen with viable sperm was found to intermittently drain through a fistulous tract that opened to the scrotum [9]. Another vasocutaneous fistula case report from the 1980s described a vasocutaneous fistula to the scrotum secondary to sperm granuloma formation [10]. From a review of the literature, fistula drainage or abscess drainage following vasectomy has primarily been located in the scrotum. We describe an unusual, delayed presentation of an anal fistula following a bilateral vasectomy procedure that was complicated by scrotal and perineal infections. The anatomical location of this fistula and the timeline of its development are unique and different from other post-vasectomy fistulas that have been previously reported. The lack of fistula communication to the vas deferens and the development of the fistula after scrotal and subsequent perineal post-vasectomy infections suggests that the anal fistula may have developed following incomplete management of a post-vasectomy abscess, resulting in delayed spontaneous drainage of the latter to the perineum.

## Case presentation

A male individual in his 40s presented to our clinic six months after undergoing an uneventful bilateral vasectomy at another institution. He experienced significant scrotal pain and swelling three days after the procedure. These symptoms improved with a course of ciprofloxacin.

Approximately three months after vasectomy, the patient developed swelling and dark discoloration at the base of the scrotum and in the perineum, accompanied by the opening of a blister and some bleeding from that area. A scrotal ultrasound performed at that time (at an outside facility, prior to his presentation at our clinic, and thus not available for publication) revealed a 3.2 x 1.9 x 1.4 cm complex fluid collection in the superficial soft tissue of the posterior midline perineum, communicating with the overlying skin. This was associated with peripheral vascularity suggestive of an abscess.

**Figure 1:**
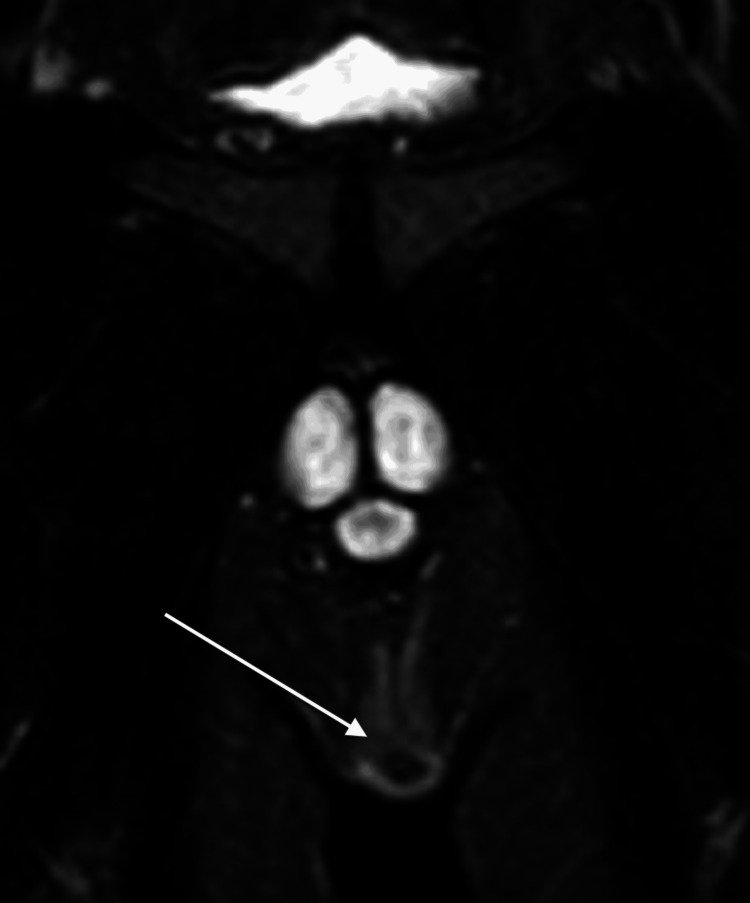
Pelvic MRI showing a small superficial collection (arrow) within the subcutaneous fat of the left perineum

The patient then underwent a pelvic MRI (Figure 1), which showed a superficial collection within the subcutaneous fat of the left perineum, measuring 1.8 x 0.5 x 1.5 cm. There was peripheral edema and hyperenhancement of the adjacent fat, possibly representing a small superficial abscess or inflamed collection. There was no visualized involvement of the scrotum. Some hyperenhancement extended posteriorly towards the anus; however, no fistula tract to the anus was observed. No intervention was performed at that time, as the physical exam prior to the planned surgery was unremarkable.

**Figure 2:**
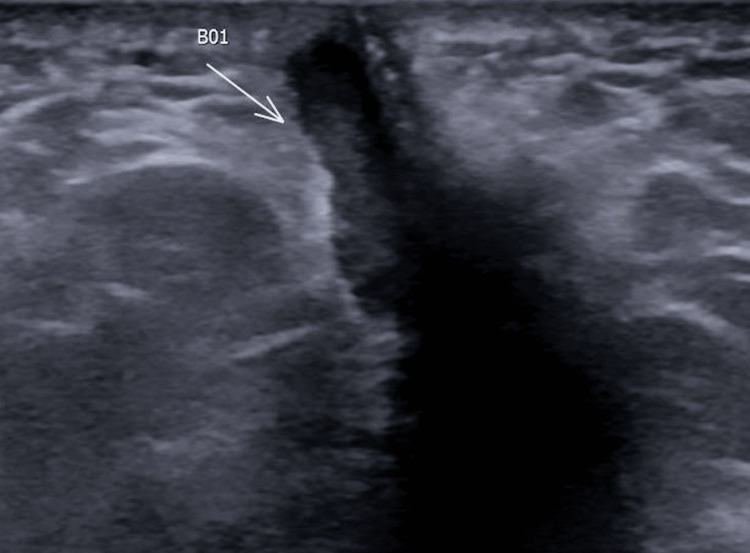
Pelvic ultrasound showing a hypoechoic, hypervascular area to the left of midline perineum (arrow) extending to the anus, measuring approximately 4.6 x 0.9 x 1.0 cm

Despite this, the perineal swelling, discoloration, and drainage continued to appear intermittently until the patient's presentation to our clinic. He denied dysuria, visible hematuria, or association of his intermittent perineal symptoms with urination. His initial evaluation at our clinic included a physical examination, which did not reveal any scrotal or perineal palpable fluid collection, drainage site, or skin discoloration. Urinalysis and scrotal ultrasound were negative, but pelvic ultrasound demonstrated a hypoechoic, hypervascular area to the left of the midline perineum extending towards the anus, measuring 4.6 x 0.9 x 1.0 cm (Figure 2). 

This finding prompted a repeat pelvic MRI, which was performed approximately two weeks later and showed resolution of the fluid collection with no evidence of abscess or fistula.

**Figure 3:**
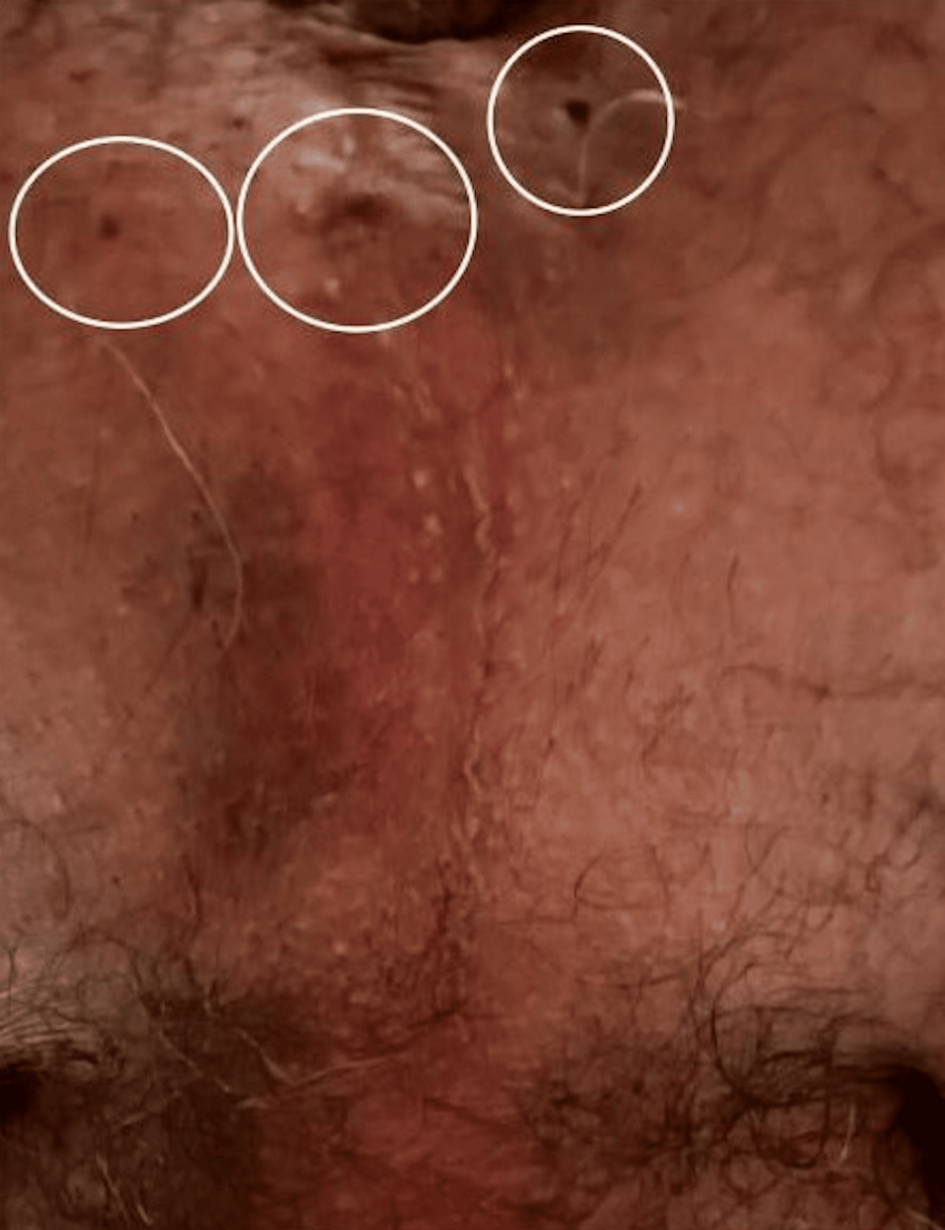
Draining perianal sites

Nevertheless, the patient’s intermittent appearance of perineal swelling, discoloration, and drainage of serosanguinous fluid continued through multiple sites (Figure 3). 

Given the perineal (rather than scrotal) location of these symptoms, the patient was referred for a consultation at the colorectal surgery (CRS) clinic. His evaluation there revealed a small wound anterior to the anus with no surrounding erythema, induration, fluctuance, or drainage. No masses or lesions were noted on rectal examination. Anoscopy showed normal mucosa, with no internal fistula opening. The patient did not have any other gastrointestinal symptoms, leading the CRS team to determine that a colonoscopy was not warranted.

A mutual decision was then made to proceed with examination under anesthesia (EUA), with possible excision of any fistulous tract, if found. EUA revealed palpable perineal firmness. Examination of the anus and distal rectum by the CRS team did not demonstrate any fistulous involvement. Careful application of a lacrimal probe was able to track approximately 1 cm adjacent to the perineal firmness; this area was unroofed with no expression of pus or fluid. Subsequently, the area was dressed with packing strips. Following this procedure, the patient’s small perineal wound gradually healed by secondary intention; however, he continued to experience intermittent episodes of perineal bleeding. He also had an episode of hematospermia, which resolved shortly and was not accompanied by visible hematuria or dysuria. Urine microscopy showed four red blood cells per high-powered field. Renal ultrasound and cystourethroscopy were both unremarkable. 

**Figure 4:**
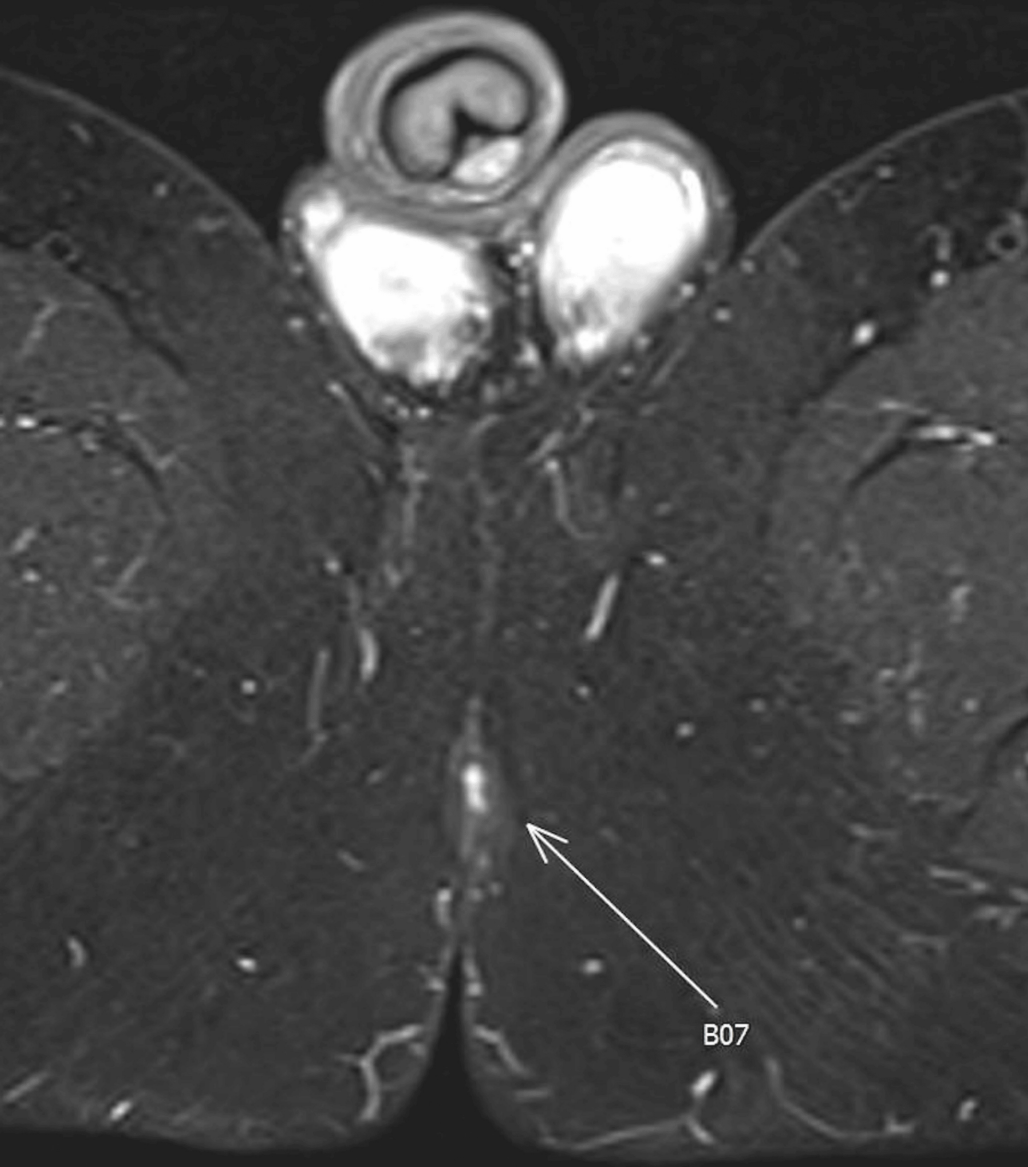
Pelvic MRI showing small sinus tract (arrow) in the perineum midline anterior to the anus

Due to the continued intermittent episodes of perineal bleeding, a pelvic MRI was repeated, which showed a small sinus tract in the perineum midline anterior to the anus without any definite internal opening into the anal canal. No evidence of fistula or abscess was visualized (Figure 4). 

**Figure 5:**
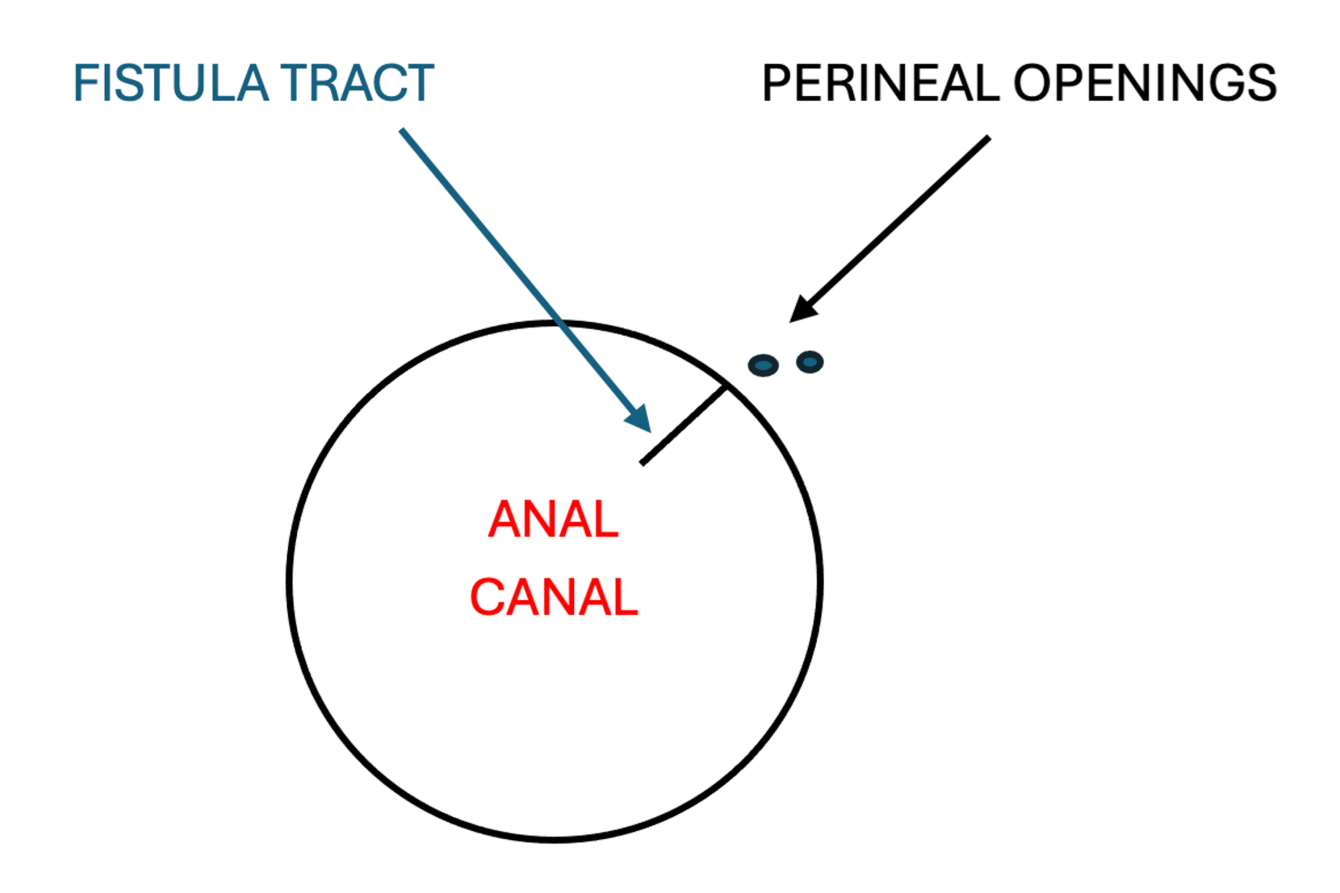
Illustration of the location of the perineal openings and the course of the fistula tract (the circle represents the anal canal) Image Credits: Author Moshe Wald

The patient was then taken again to the operating room by the CRS team. A careful anorectal examination, with the aid of operative anal retractors, revealed two small perineal openings 1-2 cm anterior to the anal canal. The openings were probed and a tract that extended inferiorly to the anal canal was identified; the instillation of half-strength hydrogen peroxide solution into the tract helped identify a small anterior anal opening at the anal verge (Figure 5). The fistulous tract was superficial, with no sphincter muscle involvement and no communication with the vas deferens. A fistulotomy was performed by incising the roof of the fistula tract over a fine probe; the tract was then debrided and hemostasis was achieved. The edges of the tract were marsupialized to prevent fistula reformation.

The patient returned for a follow-up four weeks post-operatively, at which time he was found to be recovering well, and the fistulotomy site was healing appropriately. Five months after the fistulotomy, the patient was doing well, with no additional perineal drainage, bleeding, or pain.

## Discussion

In general, infections have been reported to occur in 1-2% of men who undergo bilateral vasectomy [1]. Post-vasectomy infections typically occur at the scrotal incision sites but can, less commonly, involve the epididymis or the urinary tract. Most post-vasectomy infections can be successfully managed with a course of antibiotics. However, severe genital infections, such as Fournier’s gangrene, which require surgical intervention, as well as unusual genital infections, such as syphilis chancre formation, have been reported. Additionally, remote non-genital infections, such as endocarditis requiring valve reconstruction, have also been described [4-6].

Vasocutaneous fistula or abscess formation after vasectomy is rare and has primarily been reported in the scrotum. Only 16 cases of cutaneous fistulas to the vas deferens were reported over 70 years in a case series from the early 1990s [7]. A recent review of rare post-vasectomy complications in 2023 described cases of vasocutaneous fistula with retrograde urine flow through the ejaculatory duct, seminal vesicles, and vas deferens, eventually draining through the scrotal skin [8]. These fistulas developed in patients with a recent surgical history of TURP or neurogenic bladder or bladder outlet obstruction [8]. Two case reports detailed the formation of a post-vasectomy fistula in the scrotum. In one case, semen containing viable sperm was found to intermittently drain through a fistulous tract that opened to the scrotum [9]. In the other case, the formation of a scrotal vasocutaneous fistula was attributed to a previous development of post-vasectomy sperm granuloma [10].

Here, we describe an unusual, delayed presentation of anal fistula that developed following vasectomy. In this patient, the anal fistula may have developed following the non-surgical management of a post-vasectomy abscess, which might not have completely resolved the underlying infectious process, resulting in gradual extension of the latter towards the anus, with eventual delayed spontaneous drainage of the abscess pocket to the perineum. While no communication with the scrotum or perirectal abscess was identified during the first examination under anesthesia and unroofing of the perineal pocket, the patient did have a one-week period of hematospermia following exploration, with bloody drainage to the perineum occurring at the same time, which may suggest a possible fistulous process. While the patient’s condition initially improved following the unroofing of the abscess cavity, the subsequent recurrence of symptoms raised concerns about an undetected fistulous process and prompted a repeat pelvic MRI, which showed a small perineal sinus tract but did not demonstrate definitive internal opening into the anal canal. The fact that the anal fistula was eventually identified during a second examination under anesthesia suggests the limitations of MRI in detecting small anal fistulas and emphasizes the importance of examination under anesthesia when there is continued clinical suspicion of such a condition. 

This case presented is unusual, as typically, infectious complications following a vasectomy procedure are limited to the scrotum, resolve with treatment using antibiotics (with or without surgical exploration), and do not drain to the perineum. Furthermore, most post-vasectomy complications are minor and occur in the immediate post-procedural period rather than several months following surgery.

Rare cases of more severe complications following vasectomy, including Fournier's gangrene and delayed complications like fistula formation, have been reported in patients with an underlying voiding dysfunction, a history of TURP, or an immunocompromised state. This patient’s case is unique as he had no known predisposing risk factors and exhibited a delayed, prolonged presentation. This highlights the importance of early surgical drainage of post-vasectomy abscesses and sheds light on the rare possibility of extra-scrotal fistulas developing after vasectomy, even in patients without significant comorbidities. 

## Conclusions

We present a case of an anal fistula that developed after bilateral vasectomy. Key findings included a post-vasectomy scrotal infection, followed by recurrent, intermittent episodes of perineal drainage. This anal fistula was detected only after a second examination under anesthesia, at which time it was treated successfully with fistulotomy. Following the surgery, the patient recovered well and remained symptom-free. Anal fistula is a rare late complication of vasectomy, which apparently can occur in patients without significant comorbidities, and may be difficult to detect with MRI imaging. Diagnosis can be made through careful examination under anesthesia, and anal fistulotomy allows for complete healing and resolution of symptoms. The presumed mechanism for fistula formation, in this case, is the eventual drainage of a partially treated post-vasectomy scrotal abscess through the perineum, which underscores the importance of early surgical drainage of post-vasectomy abscesses.
